# Supplementary material for: Discrete choice experiment to investigate preferences for psychological intervention in cardiac rehabilitation
Source: BMJ Open. 2022 Nov 7;12(11):e062503. doi: 10.1136/bmjopen-2022-062503 (PMC9644324; doi:10.1136/bmjopen-2022-062503)

A discrete choice experiment to investigate preferences for psychological intervention in cardiac rehabilitation

Corresponding author: gemma.shields@manchester.ac.uk

Supplementary material

Supplementary Table 1 Preliminary conditional logit results

Attribute/level	General population sample Coef. (SE; p)	Trial sample Coef. (SE; p)	All respondents Coef. (SE; p)
**Psychological intervention to be received alongside your standard cardiac rehabilitation programme**			
Peer group support that provides non-specific support and advice	-0.012 (0.041; 0.770)	0.006 (0.100; 0.952)	-0.005 (0.038; 0.893)
Group psychological therapy where you are not required to share detailed information about personal concerns/experiences	-0.111 (0.042; 0.008)*	0.039 (0.103; 0.705)	-0.091 (0.038; 0.018)*
Group psychological therapy where you may be required to share detailed information about personal concerns/experiences	-0.194 (0.045; 0.000)*	-0.106 (0.109; 0.328)	-0.189 (0.041; 0.000)*
Individual psychological therapy	0.318 (0.040; 0.000)*	0.061 (0.103; 0.552)	0.285 (0.037; 0.000)*
**The person who provides the psychological therapy**			
Occupational therapist trained to deliver psychological therapy	-0.021 (0.042; 0.610)	-0.025 (0.102; 0.806)	-0.027 (0.038; 0.487)
Cardiac rehabilitation professional trained in delivery of psychological therapy	0.074 (0.041; 0.068)	0.461 (0.102; 0.000)*	0.128 (0.037; 0.001)*
Health care professional trained in delivery of psychological intervention, no background in cardiac rehabilitation or psychology	-0.079 (0.043; 0.064)	-0.429 (0.109; 0.000)*	-0.126 (0.039; 0.001)*
Clinical psychologist	0.026 (0.042; 0.538)	-0.007 (0.103; 0.942)	0.025 (0.039; 0.516)
**The information given to you prior to accepting and starting treatment that gives you an idea of what to expect from the therapy**			
No information provided	-0.139 (0.043; 0.001)*	-0.209 (0.106; 0.049)*	-0.140 (0.039; 0.000)*
A printed leaflet of information	-0.032 (0.042; 0.454)	-0.007 (0.106; 0.951)	-0.026 (0.039; 0.502)
An overview of the therapy from a healthcare provider with a chance to ask questions	0.107 (0.041; 0.009)*	0.204 (0.100; 0.043)*	0.115 (0.037; 0.002)*
An overview of the therapy from a healthcare provider with a chance to ask questions and a printed leaflet	0.064 (0.041; 0.123)	0.012 (0.101; 0.905)	0.051 (0.038; 0.177)
**Location you need to visit to attend psychological therapy sessions**			
Primary care (GP surgery)	-0.103 (0.043; 0.016)*	0.061 (0.107; 0.567)	-0.077 (0.039; 0.051)

Community care (NHS clinic in the community)	0.106 (0.040; 0.009)*	0.035 (0.100; 0.730)	0.095 (0.037; 0.011)*
Outpatient (clinic at a hospital)	-0.038 (0.042; 0.375)	0.029 (0.103; 0.781)	-0.035 (0.039; 0.368)
Tertiary care (specialist/University hospital)	0.035 (0.043; 0.406)	-0.124 (0.106; 0.239)	0.017 (0.039; 0.662)
**Additional cost to the NHS**			
NHS cost	-0.001 (0.000; 0.000)*	-0.001 (0.000; 0.000)*	-0.001 (0.000; 0.000)*
**Therapy provision**			
Alternative Specific Constant[1]	0.785 (0.042; 0.000)*	2.068 (0.165; 0.000)*	0.870 (0.040; 0.000)*

* Statistical significance (P<0.05)
[1] This constant represents people's preferences for some form of cardiac rehabilitation (specifically one with the mean effect for each of the qualitative attributes and no cost) versus receiving no rehabilitation).

Supplementary Figure 1 Swait and Louviere plot of coefficients

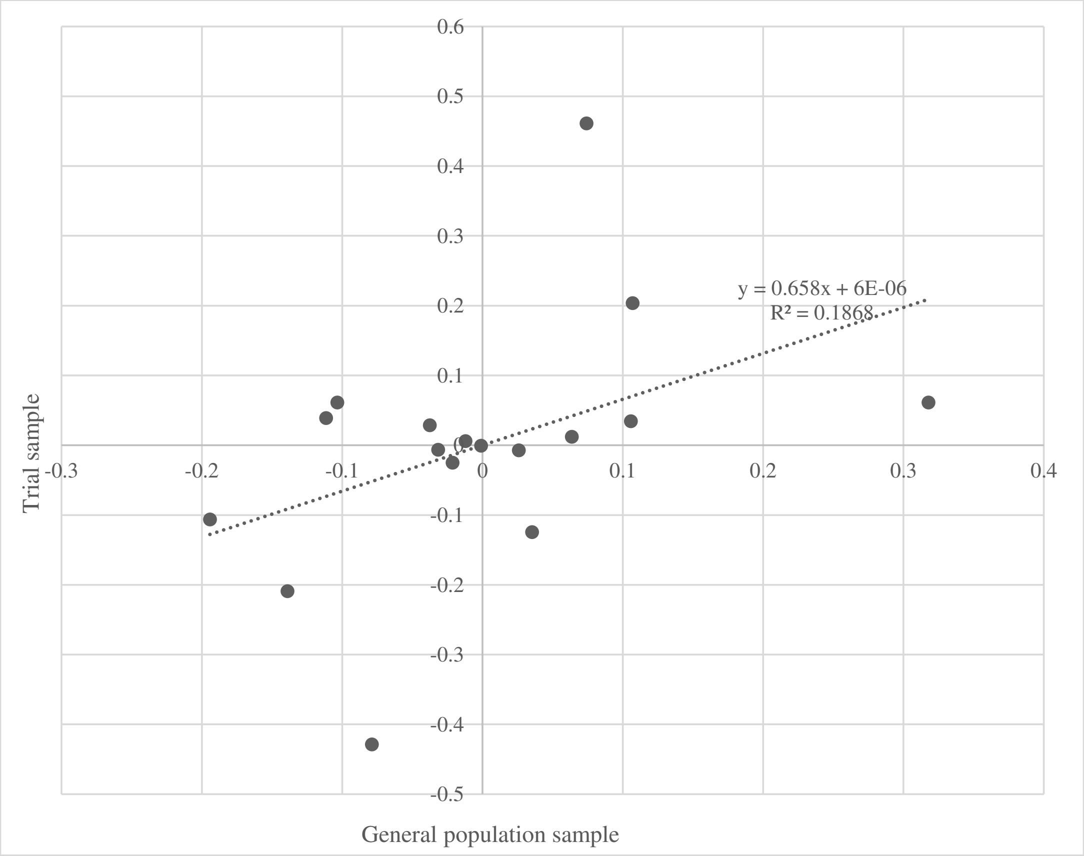

Supplement: Supplementary data [file bmjopen-2022-062503supp001.pdf]
